# Personalized alignment in total knee arthroplasty: current concepts

**DOI:** 10.1051/sicotj/2021021

**Published:** 2021-03-26

**Authors:** Sébastien Lustig, Elliot Sappey-Marinier, Camdon Fary, Elvire Servien, Sébastien Parratte, Cécile Batailler

**Affiliations:** 1 Orthopaedics Surgery and Sports Medicine Department, FIFA Medical Center of Excellence, Croix-Rousse Hospital, Lyon University Hospital 69004 Lyon France; 2 Univ Lyon, Claude Bernard Lyon 1 University, IFSTTAR, LBMC UMR_T9406 69622 Lyon France; 3 Department of Orthopaedic Surgery, Western Health 3002 Melbourne Australia; 4 Australian Institute for Musculoskeletal Science (AIMSS), The University of Melbourne and Western Health 3002 St. Albans VIC Australia; 5 LIBM – EA 7424, Interuniversity Laboratory of Biology of Mobility, Claude Bernard Lyon 1 University 69003 Lyon France; 6 International Knee and Joint Centre 46705 Abu Dhabi United Arab Emirates; 7 Institute for Locomotion, Aix-Marseille University 13009 Marseille France

**Keywords:** Total knee arthroplasty, Personalized alignment, Kinematic alignment, Restricted alignment, Functional alignment, Implant survivorship

## Abstract

Traditionally in total knee arthroplasty (TKA), a post-operative neutral alignment was the gold standard. This principle has been contested as functional outcomes were found to be inconsistent. Analysis of limb alignment in the non-osteoarthritic population reveals variations from neutral alignment and consideration of a personalized or patient-specific alignment in TKA is challenging previous concepts. The aim of this review was to clarify the variations of current personalized alignments and to report their results. Current personalized approaches of alignment reported are: kinematic, inverse kinematic, restricted kinematic, and functional. The principle of “kinematic alignment” is knee resurfacing with restitution of pre-arthritic anatomy. The aim is to resurface the femur maintaining the native femoral joint line obliquity. The flexion and extension gaps are balanced with the tibial resection. The principle of the “inverse kinematic alignment” is to resurface the tibia with similar medial and lateral bone resections in order to keep the native tibial joint line obliquity. Gap balancing is performed by adjusting the femoral resections. To avoid reproducing extreme anatomical alignments there is “restricted kinematic alignment” which is a compromise between mechanical alignment and true kinematic alignment with a defined safe zone of alignment. Finally, there is the concept of “functional alignment” which is an evolution of kinematic alignment as enabling technology has progressed. This is obtained by manipulating alignment, bone resections, soft tissue releases, and/or implant positioning with a robotic-assisted system to optimize TKA function for a patient’s specific alignment, bone morphology, and soft tissue envelope. The aim of personalizing alignment is to restore native knee kinematics and improve functional outcomes after TKA. A long-term follow-up remains crucial to assess both outcomes and implant survivorship of these current concepts.

**Nomenclature**
CT scan: Computerized Tomography scan
HKA: Hip Knee Ankle angle
KA: Kinematic Alignment
LDFA: Lateral Distal Femoral Angle
LTR: Lateral Tibial Resection
MA: Mechanical Alignment
mFA: Mechanical Femoral Axis
MPTA: Medial Proximal Tibial Angle
mTA: Mechanical Tibial Axis
MTR: Medial Tibial Resection
OKS: Oxford Knee Score
PCA: Posterior Condylar Axis
STE: Surgical Trans Epicondylar axis
TKA: Total Knee Arthroplasty
TJL: Tibial Joint Line

## Introduction

Traditionally in total knee arthroplasty (TKA), a post-operative neutral alignment was a standard principle [1–3]. To obtain a mechanical alignment the femoral and tibial components are positioned at 90° to the tibial and femoral mechanical axis. This alignment philosophy for knee arthroplasty was driven by equalizing load on the implant to decrease wear and loosening rather than restoring normal knee kinematics and function. Mechanical Alignment (MA) in TKA has demonstrated good long-term implant survival [2, 4, 5]. However, functional outcomes of the TKA are inconsistent. Bonnin et al. found 75%–89% of patients with TKA reported significant discomfort [6]. Discomfort during activities of daily living is a significant cause of patient’s dissatisfaction after TKA [6–8].

Several recent studies have described limb alignment in non-osteoarthritic and osteoarthritic populations. A systematic review by Moser et al. reported that the mean hip knee ankle angle (HKA) ranged from 176.7 to 180.7° in a native non-osteoarthritic knee [9]. The majority of studies in the review (12–15) did not report a neutral native limb alignment of 180°, apart from Hovinga and Lerner [10] or Khattak et al. [11]. The coronal alignment variability in non-osteoarthritic knees raises the question of a limb alignment of 180° is “normal”. This alignment could be not the target in TKA for all patients. Hess et al. in a second paper reviewed femorotibial alignment in osteoarthritic knees and concluded there were a large variation in overall coronal limb alignment as well as isolated tibial and femoral coronal alignments [12]. This observation continues to fuel the discussion and classification of limb alignment. In an asymptomatic Cohort of 250 adults, Bellemans et al. described a neutral alignment as 180° ± 3°, constitutional varus inferior to 177°, and constitutional valgus superior to 183° [13]. Hirschmann et al. in more recent studies further classified the HKA alignment to include the femoral and the tibial mechanical angles (FMA and TMA, respectively) [14–16]. This classification is more useful and is an explanation of how current concepts of alignment variations in both femoral and tibial cuts will affect the final alignment.

As the concept of MA was questioned in the 1980 s anatomical alignment was described by Krackow and Hungerford with the aim to improve functionality by closer reproducing the native knee alignment [1, 17], but the alignment was similar for all and not personalized. This lead to the development of several concepts of personalized alignment: kinematic, inverse kinematic, restricted kinematic, and functional. The distinction between these different concepts of alignment is sometimes difficult to interpret and reporting inconsistent in the literature.

The goal of this current concepts paper is to clarify the different types of current personalized alignments, summarize their main principles and report their results.

## Kinematic alignment

### Principles

**Figure 1:**
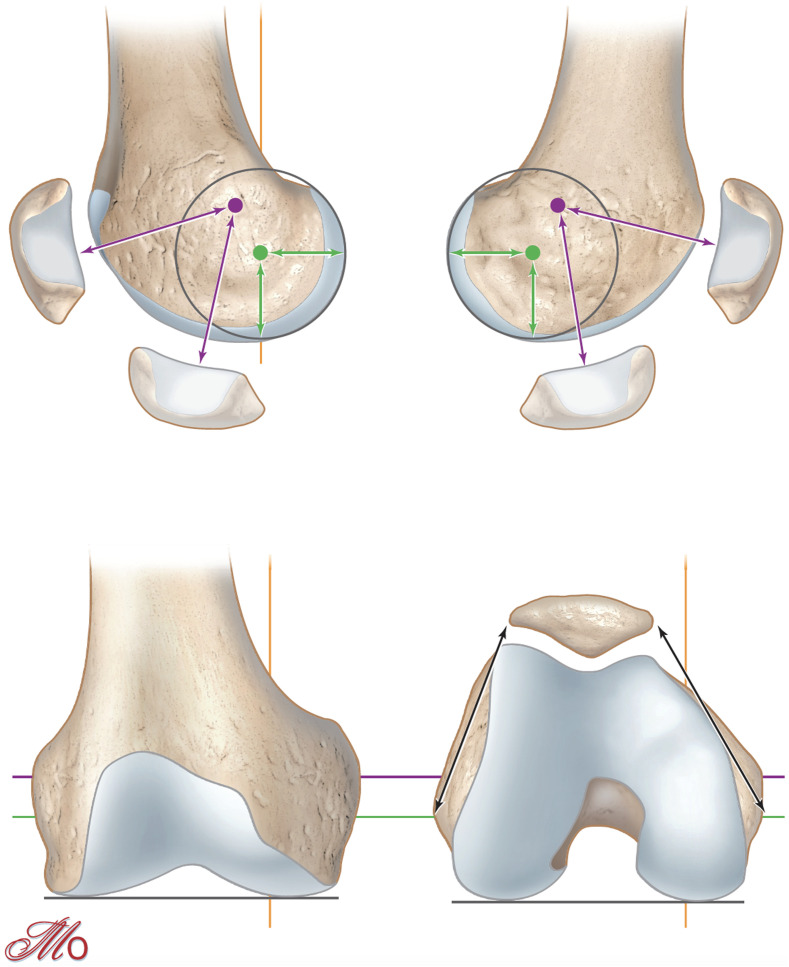
The femoral transverse axis about which the tibia extends and flexes is the most distal and posterior (Green line). The femoral transverse axis about which the patella extends and flexes is more proximal and anterior (Violet line). The longitudinal axis about which the tibia externally and internally rotates on the femur passes through the medial femorotibial compartment (Yellow line). All three axes are either perpendicular or parallel to the joint lines (Blackline).

Kinematic alignment (KA), described by Howell et al. in 2006, is an “individualized” or patient-specific technique [18]. The aim of KA is knee resurfacing with restitution of the pre-arthritic anatomy and preservation of the soft-tissue envelope. In this technique the knee is represented in three kinematic axis with respect to the joint lines of the posterior and distal femur (Figure 1): one transverse axis in the femur about which the tibia extends and flexes, one about which the patella extends and flexes and one longitudinal axis about which the tibia externally and internally rotates on the femur. All three axes are either parallel or perpendicular to the joint lines [19]. By resurfacing the femorotibial joint, the KA technique aims to co-align the axes and joint lines of implants with the three “kinematic” axes and joint lines of the native joint. The surgeon resurfaces the femur maintaining the pre-arthritic femoral joint line obliquity, and adjusts the extension and flexion gaps with the resection of the proximal tibia. Sometimes, KA involves complex algorithms to balance the extension and flexion gaps [20]. The tibial compensation can result in more oblique tibial varus resections with an increased medial tibial cut compared to MA.

Tibial and femoral resection thicknesses are validated with caliper measures and must match the thickness of the implants after compensating for saw cut and wear. It restores pre-arthritic ligament lengthening, does not create gap imbalance, minimizes the need for release [21–24]. Howell does not place restrictions on the patient’s anatomy or final correction. For this reason, KA requires an accurate surgical technique and can be performed by multiple methods: conventional instrumentation, computer navigation, personalized instruments, or robotic-assisted.

### Surgical technique

**Figure 2:**
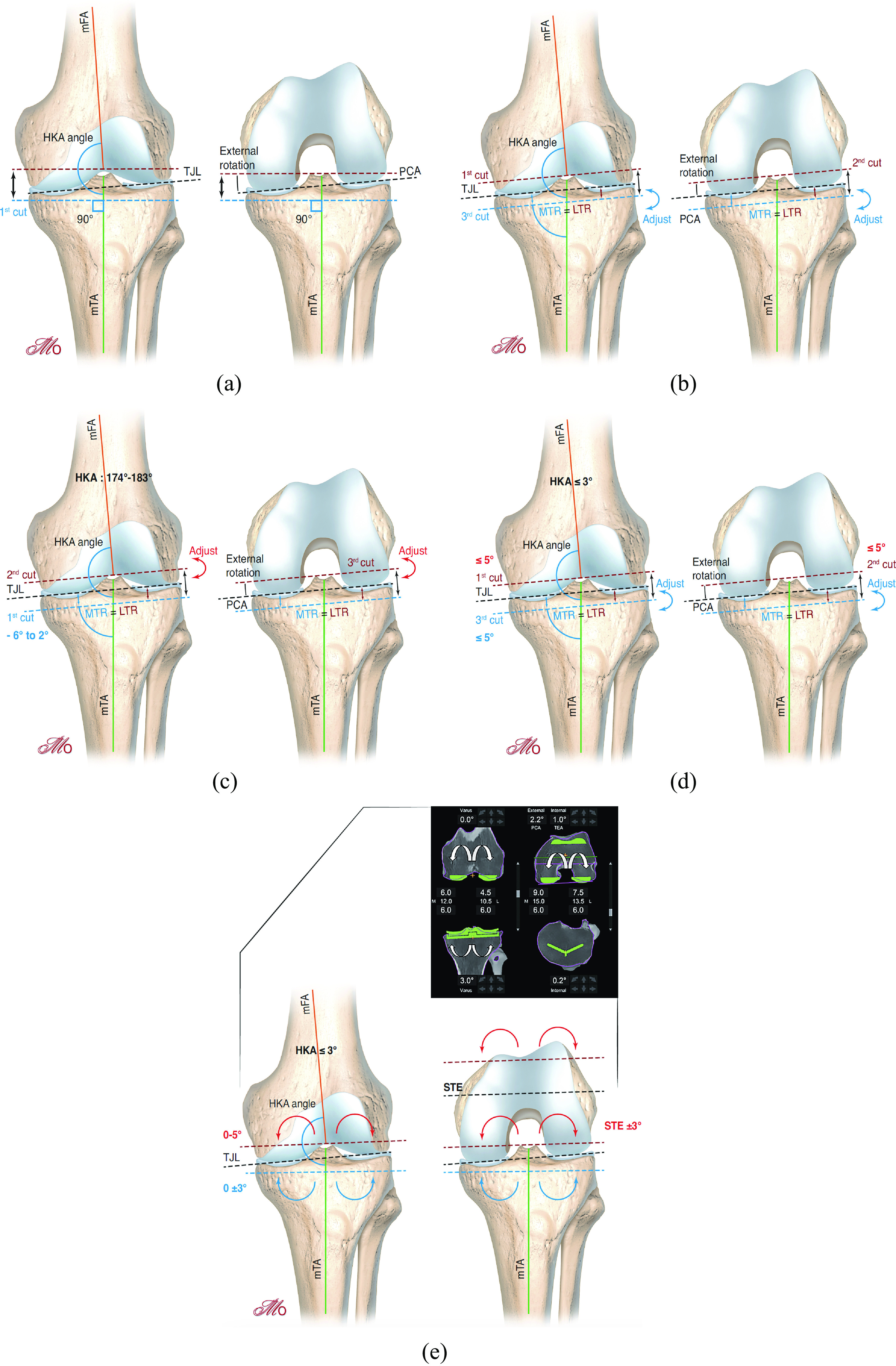
(a) Mechanical alignment, (b) kinematic alignment, (c) inverse kinematic alignment, (d) restricted kinematic alignment, (e) functional alignment.

KA implantation is usually a measured resection technique with the femur first (Figure 2b). Initially, the surgeon must estimate the individual physiological knee laxity throughout the range of motion of the knee and the amount of bone loss. The first cut is the distal femoral cut which is parallel to the joint line after correcting for the estimated bone loss. The posterior femoral cut is then performed parallel to the posterior condylar plane (usually no wear posteriorly). Resection of bone (corrected for wear) from the posterior and distal femur is equal in thickness to the femoral implant condyle which kinematically aligns the femoral implant. The surgeon then cuts the tibia parallel to the joint line. The tibial resected bone (corrected for wear) is equal in thickness to the tibial component will kinematically align the tibial component [19].

The surgeon must always measure each bone resection with a caliper. The thickness of the bone cut is calculated by deducting 1 mm from the component thickness for the saw cut thickness and by estimating the amount of articular surface wear. The cartilage thickness is frequently almost 2 mm on the distal and posterior parts of the femoral condyles.

During the trials, if there is a femorotibial soft-tissue imbalance (tightness, excessive laxity) and the soft-tissue envelope remains intact (no release/deficiency), the proximal tibia should be recut to compensate. Kinematic femoral component implantation is relatively straightforward and highly reproducible compared to a kinematic tibial cut and component insertion. A common technique for this method is to use personalized (patient-specific) cutting guides that enable additional degrees of slope/valgus/varus. In summary, the ligament balancing is performed by the bone cuts and adjusted as required by the tibial cut. This results in two important limitations that can occur with KA and have led to the development of restricted KA and inverse KA which is discussed later.

### Results

Sappey-Marinier et al. performed a systematic review of the clinical and radiological outcomes after TKA with KA versus with MA at 2 years of follow-up [25]. They reported that four of five prospective randomized controlled trial studies did not find any difference between the two groups (MA or KA) for all scores [26–29]. One study reported that kinematically aligned TKA had significantly better scores for a range of motion, function, pain than those who underwent mechanically aligned TKA [30]. Young et al. [26] found no difference between kinematic alignment (*n* = 49) and mechanical alignment (*n* = 50) in Oxford Knee Score (OKS) (42 ± 6 and 41 ± 6, respectively) at 2-years follow-up. A randomized controlled trial by Dossett et al. [30] revealed a significant difference (*p* = 0.005) with KA outcomes (*n* = 44) greater than MA (*n* = 44) in OKS (40 ± 10.2 and 33 ± 11.1, resp.) at 2-years follow up. Of note, 90% of knees in the latter study were preoperatively in varus alignment and at 24 months there was no differences concerning the complication and revision rates, postoperative gait analysis, and tibial component migration.

Shelton et al. have assessed the functional outcomes and satisfaction rate of patients treated with a kinematically aligned TKA that had a contralateral MA TKA [31]. 83% of patients were satisfied with the mechanically aligned TKA when they were treated with the kinematically aligned TKA and 92% were satisfied with the KA TKA at the last follow-up. The median forgotten joint score (FJS) for KA TKA was higher than MA TKA by a significant difference of 15 points (*p* = 0.006). 56% favored the KA TKA, 8% favored the mechanically aligned TKA, and 36% rated both knees the same (*p* < 0.001). 74% of patients favored the recovery of the kinematically aligned TKA, 6% favored the recovery of the mechanically aligned TKA.

A concern with kinematic alignment is the risk of developing aseptic loosening due to the increased varus alignment. Howell et al. reported implant survival of 220 (unrestricted) KA TKA at 10 years of follow-up of 97.5% for revision for any reason and 98.4% for aseptic failure [23]. Tibial implant loosening occurred in 1 patient, with a reverse tibial slope. Using MA criteria, the percentage postoperatively aligned in the varus (valgus) outlier range (>3°) was 78% (0%) for the tibial implant, 31% 220 (5%) for the femoral implant knee, and 7% (21%) for the HKA (unknown mean varus).

## Inverse kinematic alignment

### Principles

A limitation of correcting ligament balancing with a tibial recut is that the “resurfacing” of the femur is at the expense of adjustment with the tibial cut. Two difficulties can occur if a tibial recut is necessary for ligament rebalance. Firstly, a more oblique and deeper recut will sacrifice medial tibial bone stock. Sappey-Marinier has demonstrated that an increased tibial resection depth is associated with significantly greater laxity in valgus between 30 and 90° of flexion, particularly with a tibial resection ≥ 14 mm [32]. Increasing the tibial resection could jeopardize the medial collateral ligament and could complicate TKA revision if required. The risk of early loosening with tibial secondary displacement is increased with a severe varus tibial alignment [33]. The second difficulty concerns gap balancing where an increased tibial recut impacts the flexion and the extension gaps. In the majority of “standard” cases the difference between gaps is small. But in complex cases where the recut may be asymmetrical, it could lead to laxity.

The principle of the “inverse kinematic alignment” is to “resurface” the tibia with similar medial and lateral resections after correcting for wear, maintaining the pre-articular tibial joint line obliquity. The gap balancing is then performed by adjusting the femoral posterior and distal resections (Figure 2c). This technique could avoid tibial over resection and tibia-related complications postoperatively. This technique has the advantage to manage independently the flexion and extension gaps. However, to perform an inverse KA accurately with conventional instrumentation or patient-specific guides is challenging and complex while a robotic-assisted system enables intraoperative planning of bone resections and gap balancing before the cuts.

### Surgical technique

Winnock de Grave et al. described this new concept and technique with a robotic-assisted system [34]. The tibial implant is positioned first with resection of equal amounts of bone lateral and medial on the tibia, after correcting for bone wear. The goal is to restore the native medial proximal tibial angle, within a safe zone of 84–92°. The tibial slope is determined by the pre-arthritic medial tibial slope. On the femoral side, the femoral implant is positioned to restore the medial joint line height both in flexion and extension. The extension and flexion gaps are balanced by adjusting the posterior and distal femoral resection levels. For the flexion gap, the goal is to achieve with the robotic-assisted system residual laxity of 1–3 mm in the lateral compartment and 1–2 mm in the medial compartment. For the extension gap, the goal is to achieve with the robotic-assisted system residual laxity of 1–2 mm in the two compartments. The target for the HKA angle remains in a safe zone between 174 and 183°. Readjustment of the femoral cuts a second time after the first cuts after trialing is difficult with a conventional resection guide. The robotic-assisted system estimates gap balancing prior to the cuts but also to potentially estimate and perform an adjustment and recuts after initial resections and trial.

### Results

Only Winnock de Grave et al. have reported the outcomes of the inverse KA. They found no significant difference in clinical results at 12 months between inverse KA and adjusted MA [34]. They reported a higher rate of satisfaction and significant improvement in postoperative OKS for restricted inverse KA, compared to adjusted MA. Of note, knees with preoperative varus deformity had an apparent improved functional score and satisfaction for restricted inverse KA compared to adjusted MA. No complication or revision was reported in both groups in the short term. However, these early results require further studies with increased patients and longer follow-up.

## Restricted kinematic alignment

### Principles

KA without restriction remains controversial due to the increased stress on the implants as the knee deformity increases and alignment deviates from MA increasing the risk of aseptic loosening. Nakamura et al. with finite element analysis assessed the tibiofemoral contact force in relation to the limb alignment [35]. In the varus knees, KA increases the contact stress on the tibial insert, medial tibial cortex, and bone resection surface. For moderate (10°) and severe (15°) varus knees, the maximum stress in kinematically aligned TKA increased by 24.8 and 32.2%, compared with to mechanically aligned TKA.

To account for the increasing stress, Vendittoli recommended “safe zones” for TKA alignment. He purposed a restricted KA protocol [36]. Advanced osteoarthritic knee anatomy is very variable and to avoid reproducing extreme anatomy, the restricted KA is a hybrid option between MA and KA. The algorithm involves modifications of bone cuts within a “safe range” defined by some criteria: independent femoral and tibial cuts must be within ± 5° of the mechanical axis and the HKA angle must fall within ± 3° of neutral. But the restricted KA technique follows the main technical principle of the KA technique, which is to respect as much as possible the KA of the femoral implant, and adjustment of the coronal limb alignment and joint line obliquity is first performed by adjusting the tibial implant cut.

### Surgical technique

**Figure 3:**
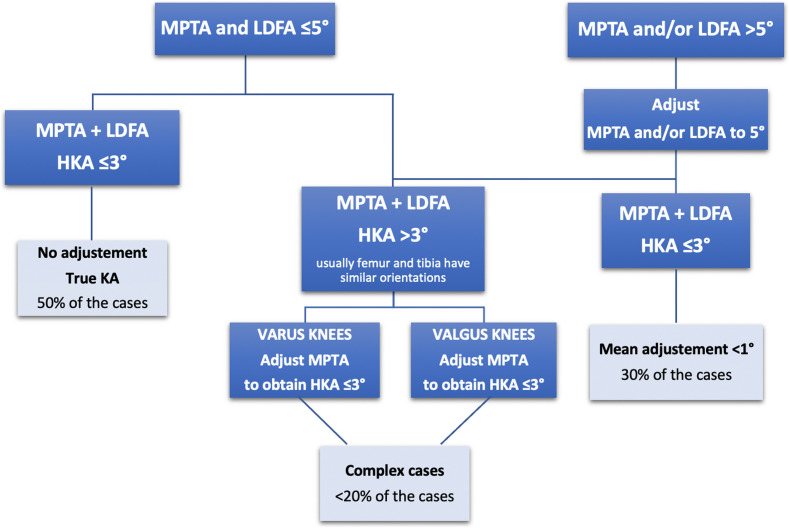
Restricted kinematic alignment protocol.

The surgical planning is well described by Vendittoli (Figure 3). There are two situations: either the tibial and femoral mechanical axis are inferior or equal to 5°, or superior to 5°.

In the first case with femoral and tibial axis inferior to 5°, if the femorotibial axis (HKA angle) is equal or inferior to 3°, the surgeon can perform the TKA with a KA technique. If the femorotibial axis is superior to 3° of varus, the tibial varus will be reduced until the HKA is equal to 3° of varus. If the femorotibial axis is superior to 3° of valgus, the tibial varus will be reduced until the HKA is equal to 3° of valgus.

In cases where the femoral and tibial axis is superior to 5°, the surgeon will correct the tibial and/or the femoral bone cuts to stay within the 5° limit. This will correct the overall HKA to within ± 3° of neutral. If the patient maintains an HKA superior to 3°, the surgeon will further adjust the tibial cut as in the first situation.

We prefer to modify the tibia to preserve as much as possible the native femoral anatomy and the flexion axis, as in the KA technique. Releases of the ligaments are not needed in cases with anatomic modifications inferior to 3°. In larger corrections, minimal releases can be added (to a much lesser degree compared to MA).

As with the unrestricted KA technique, it is important to measure the bone resections after each cut. Computer navigation and robotic-assisted systems facilitate intraoperative operative adjustment in complex cases.

### Results

Of 2475 TKA cases Almaawi et al. reported 49% required restricted KA TKA and 51% unrestricted KA TKA [36]. Blakeney et al. simulated the extension and flexion gaps on 1000 lower limb CT scan according to the restricted KA or MA protocols. An “imbalance” was defined as a difference between lateral and medial gaps. In extension, there were significantly fewer cases having an imbalance ≥ 3 mm with restricted KA (8.3%) versus MA (33%), and ≥ 5 mm with restricted KA (1.5%) versus MA (11%). With restricted KA, the percentage of knees with space imbalances inferior to 3 mm in both flexion and extension was 92% versus 63% with MA with posterior condylar reference and 49% with MA with trans epicondylar reference [37].

MacDessi et al. have reported encouraging results after TKA with restricted kinematic alignment in a randomized controlled trial [38]. They found that the mean intraoperative intercompartmental pressure difference at 10° of flexion in the kinematic group was significantly lower than in the mechanical group, using an interoperative pressure sensor. Overall, participants in the kinematic group were more likely to obtain optimal knee balance (80% vs. 35%).

Currently, no study has assessed the mid- or long-term clinical outcomes after restricted KA TKA.

## Functional alignment

### Principles

Functional alignment has similar aims and was developed for similar reasons as KA [39, 40]. It constitutes an evolution and increased precision of the KA concept. Patient-specific implants and 3D printed cutting blocks were used pre-operatively to achieve KA in total knee arthroplasty. Functional alignment is obtained by manipulating alignment, bone resections, fine-tuning component positioning, and/or soft tissue releases at the surgeon’s discretion intraoperatively with robotic-assisted systems to achieve balanced extension-flexion gaps and soft tissue tension while maintaining the patient’s native alignment. These new and constantly improving technologies enable quantifiable measurement and precision adjustment of femoral and tibial cuts, implant positioning, or tissue release in three planes, of one or two degrees, to obtain optimal functional alignment. The precision offered by robotic assistance may make achieving non-neutral alignment targets more reproducible [41], reducing the risk of missing the target and producing significant outliers of the limb alignment. Theoretically, functional TKA reduces the need for periarticular soft-tissue releases if not desired by the surgeon while restoring the patient’s native knee kinematics

### Surgical technique

Robotic-assisted systems are constantly evolving both in hardware and software platforms and algorithms. Planning may initially begin preoperatively on a 3D and be completed during the surgery prior to bone cuts. Once the bone cuts have been made and the trial is in place the robotic system, soft tissue sensor or surgeon may discover a soft tissue imbalance. Adjustments can then be assessed with software 3D manipulation virtually and then recut guidance or releases performed with the robotic-assisted system if indicated.

In the coronal plane, femoral component positioning is modified from a starting point of 0° to the mechanical axis to balance the extension gap. In the sagittal plane, the femoral component is positioned to optimize the component sizing and to avoid femoral notch by flexing up to 5°. In the axial plane, the femoral implant is aligned to the transepicondylar axis with 3° of freedom to balance the flexion gap. The size of the femoral component is selected using posterior referencing with the smallest size that does not overhang the femur, notch the anterior femur, or overhang mediolateral bone edges, and avoids overstuffing the patellofemoral joint. The femoral component is positioned at the center of the mediolateral cortical bone edges, with a small lateral position if necessary. In the coronal plane, the tibial component position is aligned to the tibial mechanical axis and modified to balance extension and flexion gaps by up to 3° of varus. Valgus tibial position should be avoided. In the sagittal plane, the tibial component position is set to match the patient’s pre-arthritic posterior tibial slope, modified to balance the flexion gap if necessary. In the axial plane, the tibial component is positioned using the line of Akagi.

The aim of functional alignment is to position the implants in the position that least compromises the knee ligaments envelope in 3D and hence to restore the obliquity and plane of the joint to that which the ligaments dictate. If the deformities are fixed, the soft-tissues release is required to balance the gaps, although the extent and frequency of such releases are smaller when compared with the MA technique.

### Results

Several studies assessed the accuracy and the reproducibility of robotic-assisted surgery [42–46]. Sires and Wilson performed CT scans postoperatively to assess the precision of the image-based robotic-assisted TKA and found that 93% of the surgical measurements were ≤ 3° of the CT measures postoperatively [46]. The use of preoperative CT scanning and the planning accuracy of robotic-assisted TKA resulted in well-balanced knees [47]. Nevertheless, no study has assessed the functional and clinical outcomes of this alignment technique, nor the implant survivorship.

## Conclusion

Several concepts and evolving surgical techniques continue to develop personalized alignment in TKA. Personalized alignment aims to restore native knee alignment and improve functional outcomes after TKA. New technologies have increased the ability to restore native knee kinematics with TKA. A long-term follow-up is crucial to determine clinical outcomes and implant survivorship of these current alignment concepts.

**Table 1:** Surgical parameters for each kind of alignment.

		Mechanical alignment	Kinematic alignment	Inverse kinematic alignment	Restricted alignment	Functional alignment
Femoral component	Flexion	Follows distal femoral bowing	Follows distal femoral bowing	Follows distal femoral bowing	Follows distal femoral bowing	Follows distal femoral bowing
		Target: 0 to 5° of flexion	Target: 2 ± 3°	Target: 2 ± 3°	Target: 2 ± 3°	Target: 0 to 5° of flexion
	Distal cut	Systematic and perpendicular to the femoral mechanical axis	Parallel to the distal femoral joint line (considering wear)	Parallel to the distal femoral joint line (considering wear)	Correct to < 5°, then Parallel to the distal femoral joint line (considering wear)	Parallel to the distal femoral joint line (considering wear)
		Target: 0°			Target: < 5°	Target: 0 to 5°
	Posterior cut	External or neutral rotation relative to posterior condylar line.	Parallel to the posterior condylar line	Parallel to the posterior condylar line	Parallel to the posterior condylar line	Surgical trans epicondylar axis; ± 3°
		Measured resection or gap-balancing techniques.				
		Posterior or anterior referencing techniques				
	Mediolateral	Slightly lateralized	Centered on the notch	Centered on the notch	Centered on the notch	Centered on the distal femur
Tibial component	Coronal cut	Systematic and perpendicular to the tibial mechanical axis	Parallel to proximal tibial joint line (considering wear)	Parallel to proximal tibial joint line (considering wear) within safe zone of 84° to 92°	Correct to < 5°, then Parallel to proximal tibial joint line (considering wear)	Perpendicular to the tibial mechanical axis
		Target: 0°	Target: −6° to 9°	Target: −6° to 2°	Target: < 5°	Target: 0 ± 3°
	Slope	Systematic. Between 2° and 7° relative to sagittal tibial mechanical axis	Parallel to the medial plateau slope	Parallel to the medial plateau slope	Parallel to the medial plateau slope	Parallel to the medial plateau slope; Target: 0° to 3°
	Rotation	Towards the medial third of the tibial tuberosity	Parallel to lateral plateau long-axis	Parallel to lateral plateau long-axis	Parallel to lateral plateau long-axis	0 to 5° of external rotation to Akagi’s line
Knee balancing		Soft tissues	Tibial cut	Femoral cut (distal and/or posterior)	Tibial cut + Soft tissues	Femoral and tibial positioning + Soft tissues
Soft tissue Release	Femorotibial joint	Frequent	None	None	Sometimes	Sometimes
	Lateral retinaculum	Sometimes	Rarely	Rarely	Rarely	Rarely
Technologies		All	All	Robotic-assisted	All	Robotic-assisted

## Conflict of interest

SL: Consultant for Stryker, Smith Nephew, Heraeus, Depuy Synthes; Institutional research support from Groupe Lepine, Amplitude; Editorial Board for Journal of Bone and Joint Surgery (Am).

ESM, CB: declare that they have no conflict of interest.

CF: Consultant for Zimmer Biomet.

ES: Consultant for Corin.

SP: Royalties for Zimmer Biomet and Newclip; Consultant for Zimmer Biomet; Treasurer for European Knee Society.
